# tsiR: An R package for time-series Susceptible-Infected-Recovered models of epidemics

**DOI:** 10.1371/journal.pone.0185528

**Published:** 2017-09-28

**Authors:** Alexander D. Becker, Bryan T. Grenfell

**Affiliations:** 1 Department of Ecology and Evolutionary Biology, Princeton University, Princeton, NJ, United States of America; 2 Woodrow Wilson School of Public and International Affairs, Princeton University, Princeton, NJ, United States of America; 3 Fogarty International Center, National Institutes of Health, Bethesda, MD, United States of America; Hokkaido University Graduate School of Medicine, JAPAN

## Abstract

**tsiR** is an open source software package implemented in the R programming language designed to analyze infectious disease time-series data. The software extends a well-studied and widely-applied algorithm, the time-series Susceptible-Infected-Recovered (TSIR) model, to infer parameters from incidence data, such as contact seasonality, and to forward simulate the underlying mechanistic model. The **tsiR** package aggregates a number of different fitting features previously described in the literature in a user-friendly way, providing support for their broader adoption in infectious disease research. Also included in **tsiR** are a number of diagnostic tools to assess the fit of the TSIR model. This package should be useful for researchers analyzing incidence data for fully-immunizing infectious diseases.

## Introduction

Mathematical models coupled with statistical inference techniques allow us to compare infectious disease theory and data, shedding light on transmission estimates, vaccine control strategies, and predicting future trends [1, 2]. These models (and inference methods) cover a spectrum from very simple (based on well-mixed, population-level assumptions) to highly complex representations (in which individual variation is modeled explicitly) [1, 3]. Even the simplest of such non-linear models can display very rich, elaborate, and potentially chaotic, dynamics [4–6].

One of the simplest and most powerful of epidemic models is the family of mass-action formulations based on the Susceptible-Infected-Recovered (SIR) equations [1, 7]. The SIR model assumes a well-mixed population, and in the most basic form balances demographic processes (e.g., births, *B*, and deaths, *μ*) with infectious disease specific properties such as contact rate, *β*, and infectious period, inverse *γ*, for a single pathogen. In general, and especially for the prototypical example, measles, the transmission coefficient, *β*, varies seasonally. The deterministic skeleton of the SIR model is shown in Eq 1; λ is the force of infection, typically defined as $\frac{\beta I}{N}$. In addition, this model requires that immunity post-infection is life-long, although this assumption can be relaxed via a Susceptible-Infected-Recovered-Susceptible (SIRS) model.

$$\begin{array}{ll} \frac{dS}{dt} & =B-\frac{\lambda S}{N}-\mu S\\ \frac{dI}{dt} & =\frac{\lambda S}{N}-\gamma I-\mu I\\ \frac{dR}{dt} & =\gamma I-\mu R \end{array}$$ (1)

Despite its simplicity, calibrating the seasonally-forced SIR model against time-series data is a difficult mathematical and statistical problem, as evidenced by the extensive literature on this subject [8–13]. The desired outcome of SIR model inference is to extract parameter estimates from a given epidemic time-series for key values such seasonal variation. In terms of the data, fitting SIR-type models is a non-trivial inferential challenge for a number of reasons. First, only one state variable—the number of cases over time—is observed. Second, there is generally substantial under-reporting of disease incidence. Adding additional complexity is the fact that the model is a continuous-time process, whereas the data are generally collected on a weekly or monthly basis. While statistically robust and powerful methods (e.g., filtering based algorithms) are readily available in packages such as **pomp** [14], they may be slow to converge and may not be suitable for analyzing many (100+) time-series.

$$\begin{array}{ll} S_{t+1} & =B_{t+1}-S_{t}-I_{t+1}\\ E\left[I_{t+1}\right] & =\beta_{t+1}S_{t}I_{t}^{\alpha } \end{array}$$ (2)

An alternative, computationally inexpensive, and highly tractable approach to these problems is provided by the time-series SIR model (TSIR model), shown in Eq 2 [8]. The TSIR model relies on two main assumptions: first, that the infectious period is fixed at the sampling interval of the data (e.g., bi-weekly for measles) and that over a long enough time (e.g., 10-20 years), the sum of births and cases should be approximately equal due to the high infectivity of pathogens such as measles and other childhood infections, in the pre-vaccine era. A full description of the TSIR model and algorithm can be found in [8]. A brief qualitative description of the algorithm follows in order to provide context for the subsequent development of the **tsiR** package.

In the TSIR framework, a regression model is first fitted between cumulative cases and cumulative births. Assuming they are equal, the slope will be the reporting rate *ρ*_*t*_ and the residuals of the regression model, *Z*_*t*_ provide the shape of the susceptible dynamics, *S*_*t*_. Next, using Eq 2 and setting the expectation to the mean, the log-linear equation shown in Eq 3 can be acquired. The mean number of susceptible individuals across the time-series, $\bar{S}$, can be inferred using profile likelihood and a seasonally repeating contact rate (52 divided by the infectious period time points, e.g., 26 for measles); *β*_*t*_, can then be estimated along with a homogeneity parameter *α* using a generalized linear model (GLM). The parameter *α* describes the epidemic saturation as well as a correction factor for switching from continuous to discrete time [15, 16]

$$\begin{array}{c} \log I_{t+1}=\log\beta_{t+1}+\log\left(Z_{t}+\bar{S}\right)+\alpha \log I_{t} \end{array}$$ (3)

The TSIR method has been used very successfully to analyze a number of childhood infections such as measles, whooping cough, diphtheria, mumps, varicella, and scarlet fever, as well as multi-strain pathogens such as dengue. [6, 8, 12, 17–20]. Analysis of this model has shown both birth rate and school-term forcing to be central drivers of the pattern of epidemics and periodicity, as well as improving our ability to predict infectious disease dynamics further into the future in both small and large populations ranging from London to Iceland [8, 12].

Although the underlying model is simple, the TSIR approach provides a number of different fitting options. When reconstructing the susceptible dynamics, options range from a simple linear regression fit between cumulative cases and cumulative births, to more sophisticated approaches such as Gaussian regressions. These choices, and which variable is the dependent variable in each case, also impact both whether the reporting rate *ρ*_*t*_ is time-invariant as well as how the residuals of the regression are calculated. Once the susceptible dynamics are reconstructed, the modeler is again faced with many more choices about the log-linear model, the GLM family and link, and which parameters to estimate and which to fix (commonly, for the study of measles dynamics, *α* is fixed to be 0.97, and $\bar{S}$ is occasionally fixed as well to be 0.035 based on analysis of pre-vaccination data from the United Kingdom [6, 12, 18]). This decision process can also be implemented in a Bayesian framework, although is a computationally more extensive task. Further choices must be made when describing the distribution of the expected value in Eq 2, as well as a deciding between a completely forward prediction versus a step-ahead prediction. These options, while relatively straightforward, are cumbersome to implement especially while working with a number of time-series. Thus, while the TSIR model has been used extensively, there is a need for an open source software package which implements these options in a user-friendly way. We have developed the **tsiR** package to address this methodological challenge and facilitate a more straight-forward and widely-accessible model-fitting process.

## 1 Methods

The **tsiR** package, available on CRAN, is written completely in the **R** programming language. Package functions and a short description of their use is included at the end of this section. Package dependencies are **kernlab** [21], **ggplot2** [22], and **reshape2** [23]. All code is publicly available on GitHub (www.github.com/adbecker/tsiR). In the following section, typewriter font refers to function arguments and quotes refer to argument inputs.

### 1.1 Data

**tsiR** requires data to be a named data frame with a ‘time’ column (that can be understood by **ggplot2**—see [22] for details), and columns for ‘cases’, ‘births’ and ‘pop’ (population size). If the number of births and population size are on a different time scale than the reported cases, these data (as well as case data) must be interpolated to the generation time. When incidence data are reported weekly and demographic data (i.e., births and population size) are reported yearly, as is often the case, these individual vectors can be interpolated to the proper infectious period timescale via the **tsiRdata** function. Note that at each time point, the births must be the number of births that occur within the IP weeks, where IP is the infectious period (in weeks). For measles, this is typically taken as two weeks, however **tsiR** does not require IP to be an integer. A dataset, **twentymeas**, is included in the **tsiR** package as a list. This dataset is a list which contains biweekly data (IP=2) for twenty locations in England from 1944-1964 and was analyzed in [11].

### 1.2 Model inference and forward simulation

The main function in the **tsiR** package is **runtsir**. This function reconstructs the susceptible dynamics, fits the log-linear relationship in Eq 3, and then resimulates the TSIR model in Eq 2 forward under the fitted parameters.

An essential argument (and assumption of the TSIR model) in the **tsiR** package is that the time step is equal to the infectious period (IP), i.e. that IP is a consistent argument throughout the data. Written based on measles, in **tsiR** all functions default to IP = 2. Thus, if a different disease is being analyzed, it is key to change the IP argument throughout the function inputs. As with all **R** functions, a full description of the function can be acquired via ?runtsir. Here we describe the main arguments. The xreg argument indicates whether cumulative cases or cumulative births are on the x-axis of the regression. Thus, the options are simply ‘cumcases’ or ‘cumbirths’ with a default of ‘cumcases’. A more extensive discussion of this choice can be found in [6]. After describing the formula for the susceptible reconstruction, the regression type must be specified via the regtype argument. Here, the options are ‘gaussian’, where the kernel size is profiled to maintain a reporting rate between zero and one, and ‘lm’, ‘lowess’, ‘loess’ or ‘spline’ regression, for a linear, a lowess, a loess, and a spline regression with 2.5 degrees freedom, respectively. If the Gaussian process fails to produce a reporting rate between zero and one, **runtsir** defaults to a loess regression. A Gaussian regression, as implemented in [12], appears to produce the most robust results in both small and large populations. However, using a Gaussian regression may produce exaggerated reporting rates when there is a single outbreak that is substantially larger in size than across the rest of the time series. The aforementioned arguments will specify the shape, *Z*_*t*_, of the susceptible dynamics, *S*_*t*_. At this stage, model parameters left to estimate are *β*_*t*_, *α*, and $\bar{S}$, although the last two can be fixed as previously mentioned. Note that the number of parameters in *β*_*t*_ is dependent on IP such that the length of *β*_*t*_ is 52 divided by IP. The only exception is where IP is equal to one. In this scenario *β*_*t*_ is truncated to 26 points, each repeated twice to preserve statistical integrity. Parameters *α* and $\bar{S}$ can be fixed via alpha and sbar if desired. For the log-linear regression in Eq 3 virtually any GLM family and link can be used, although the options are essentially ‘quasipoisson’, ‘poisson’, and ‘gaussian’ where ‘poisson’/‘quassipoisson’ take a ‘log’ link and a ‘gaussian’ family takes either a ‘log’ or ‘identity’ link.

**Fig 1:**
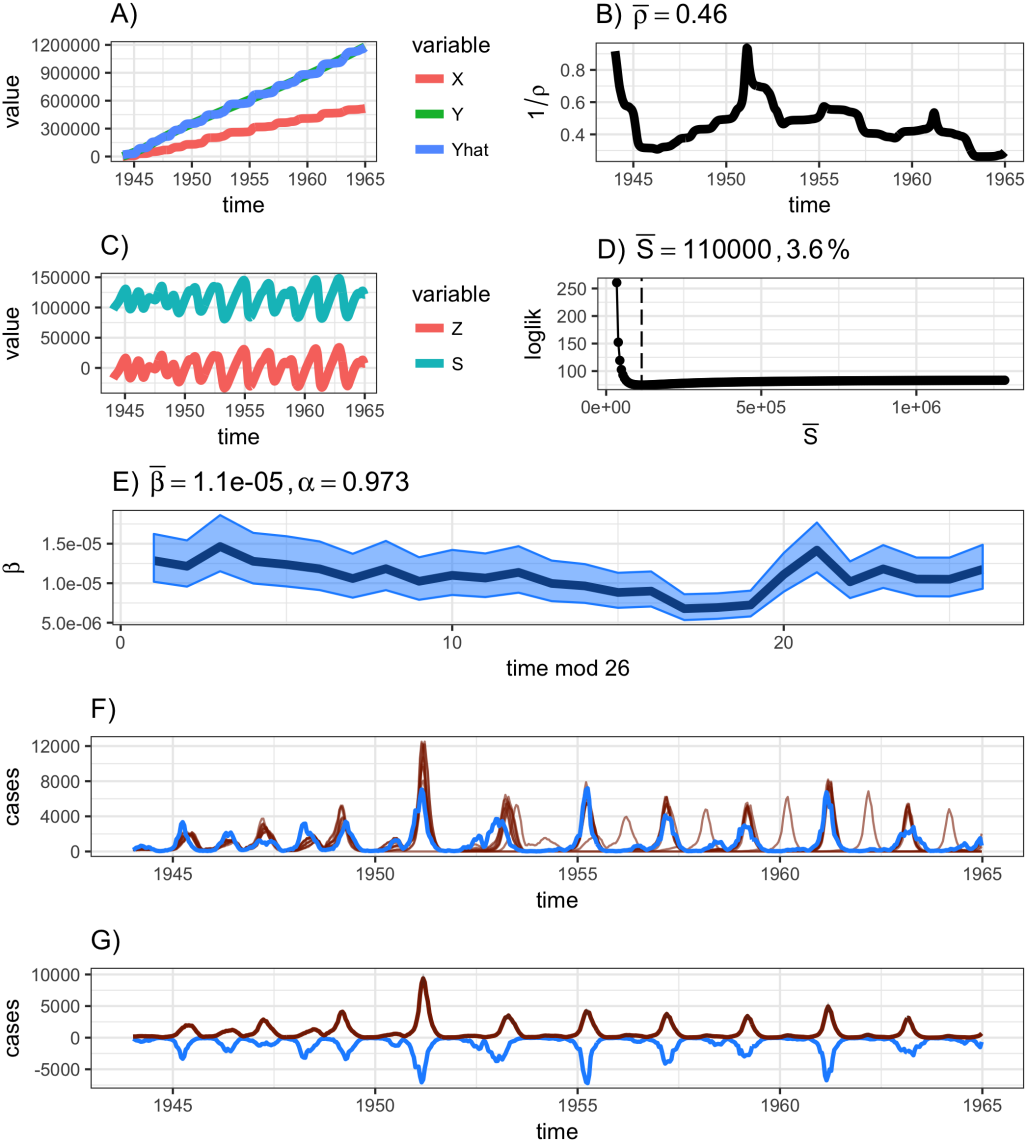
Output results from the runtsir function for London. Subplots A) and B) are the cumulative births against cumulative cases regression and estimated reporting rate, the C) and D) are the profiled $\bar{S}$ from *Z*_*t*_ and then reconstructed *S*, E) is 26-point *β*_*t*_ with the *α* and mean *β* (indicated as $\bar{\beta }$) estimate, and F) and G) are the data (blue) against 10 randomly chosen stochastic simulations (red) and the (inverse) data against mean of the simulations with confidence intervals.

At this stage, all parameters are estimated, confidence intervals have been constructed (when appropriate and the computation can be completed), and the model can be forward simulated. The **runtsir** function defaults to a full time-series ahead forward prediction, although step-ahead can be inputted as well under the pred argument. For large populations, the forward prediction can generally be simulated without fear of fade-outs (i.e., the simulation declining to zero). However, for smaller populations with frequent local ‘fade-outs’ of infection between epidemics, the forward prediction may only be able to be run a simple epidemic-ahead [12]. This constraint on the prediction model can be specified via epidemics = break as well as designating a threshold parameter that indicates when a new epidemic is started and thus where to reset the forward simulation. By default, the initial conditions (*S*_0_, *I*_0_) are ($Z_{0}+\bar{S}$, *C*_0_/*ρ*_0_) where *C*_*t*_ is cases. Thus, if *C*_0_ is zero, the forward prediction will fail. In this scenario, the data must be truncated to the first non-trivial case, or initial conditions can be fit using simple least squares per [6], although this feature could be made more robust in the future (see the Conclusions section). This can be specified using the inits.fit = T argument. Finally, the simulation distribution can be specified via method where the options are ‘deterministic’, ‘negbin’, and ‘pois’ for deterministic, Negative Binomial, and Poisson distributions. The number of simulations to perform is specified via nsim. The output of **runtsir** is a named list and can be fully plotted via the **plotres** function. The output of this model, as generated by the following code, can be seen below and is plotted in Fig 1.

LondonMeas <- twentymeas[[“London”]]

LondonRes <- runtsir(data=LondonMeas, IP = 2,

         xreg = ’cumcases’, regtype=’gaussian’,

         alpha = NULL, sbar = NULL,

         family = ’gaussian’, link = ’identity’

         method = ’negbin’, nsim = 100)

plotres(LondonRes)

**Fig 2:**
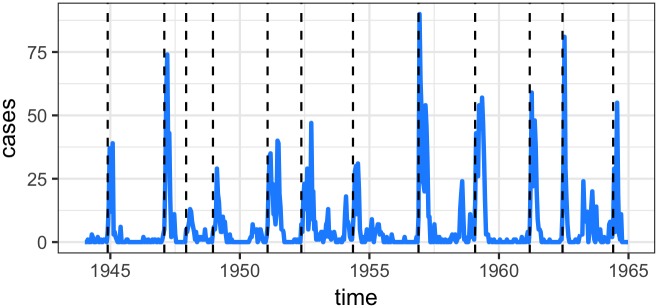
Northwich time-series data. Dashed lines are the time points in which the forward simulation resets in the epidemics = ‘break’ argument for a threshold for three.

**Fig 3:**
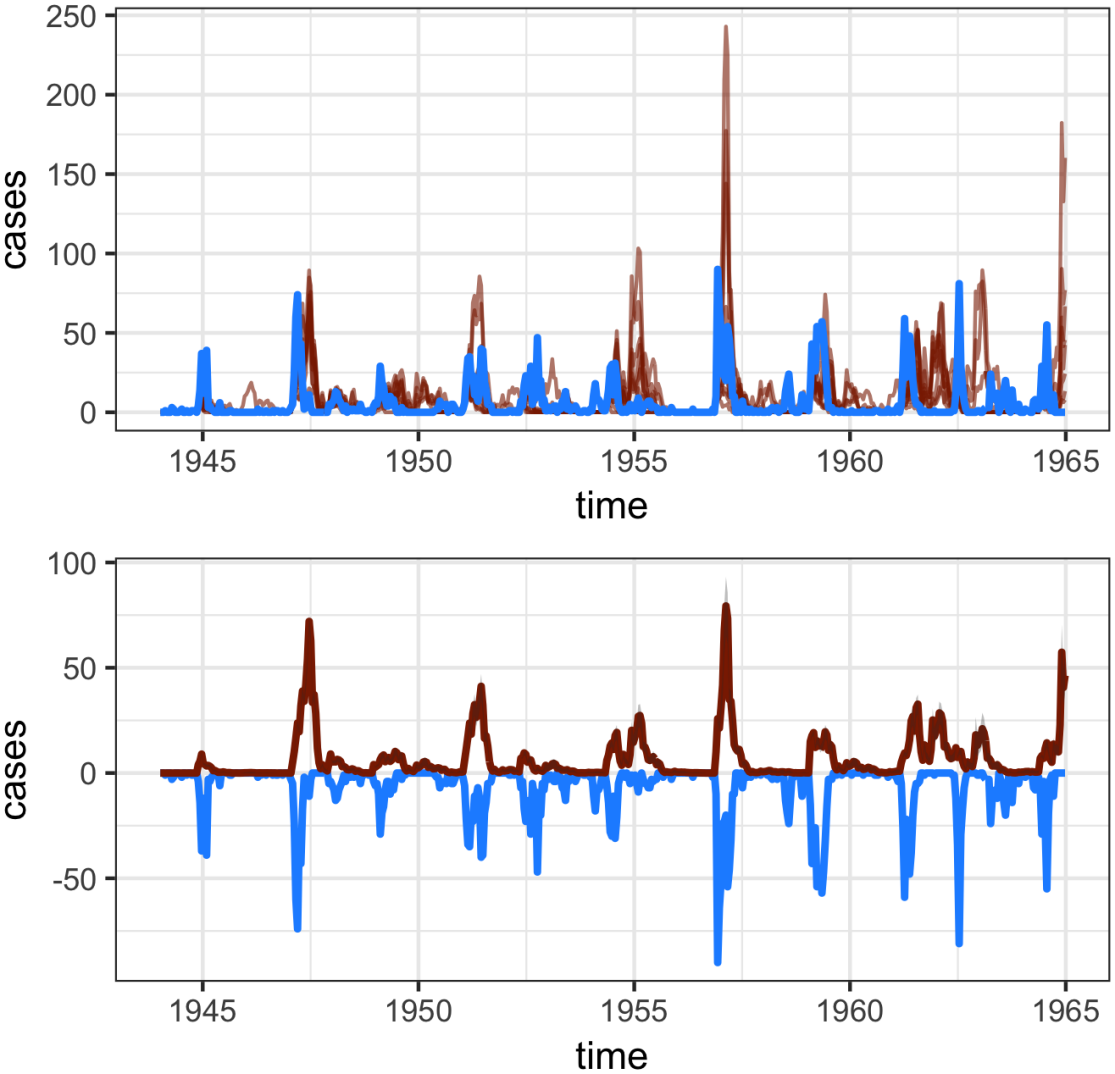
The forward simulations for the Northwich time-series data under an epidemic-ahead fit using a threshold of three. The color coding in the panels shown here are the same as in Fig 1.

The **runtsir** function is also decomposed via the **estpars** and **simulatetsir** functions. This may be more desirable if exploring a large number of simulations or analyzing sparse incidence data. In the following short example, we will examine such a data set from Northwich (Cheshire, England, population size in 1944 = 18,070). Here, we must define epidemic start and end times per [12]. Using a threshold of three, we can see where each epidemic is defined via the dashed line in Fig 2. Here, we are estimating parameters using different regression options available in **tsiR**, as well as simulating the model via a Poisson distribution and fixing *α*. Only the forward simulations are shown in Fig 3, where again the data is shown in blue and the simulation results are shown in red. Overall, the forward simulation captures the epidemic final size well for such a noisy time-series [12]. The code used to generate these fits and plots is shown below.

NorthwichMeas <- twentymeas[[“Northwich”]]

NorthwichParms <- estpars(data = NorthwichMeas, IP = 2,

            alpha = 0.97, sbar = NULL,

            regtype = ’loess’,

            family = ’poisson’, link = ’log’)

plotbreaks (data = NorthwichMeas, threshold = 3)

NorthwichRes <- simulatetsir(data = NorthwichMeas, IP = 2,

            parms = NorthwichParms,

            epidemics = ’break’, threshold = 3,

            method = ’pois’,nsim = 100)

plotcomp (NorthwichRes)

A brief summary of the main functions and their usage in the **tsiR** package follow below in Table 1. Please note for MCMC functionality, one must install **rjags** [24] independently. The MCMC functions, **mcmcestpars** and **mcmctsir**, follow generally the same arguments as their frequentist counterparts. Notable exceptions however are that a family and link no longer can be specified, and MCMC specific arguments (n.chains for the number of chains, update.iter for the number of MCMC iterations to use in the **rjags** update section, n.iter for number of MCMC iterations to perform, n.adapt for the adaptive number, and burn.in for the burn in number) can be specified. For more information on these arguments, we direct the reader to **rjags** documentation [24]. Additionally, annotated code for the London and Northwich estimations and forward simulations is included as a .R files in the Supporting Information.

**Table 1:** Summary and description of the main functions in the tsiR package.

Function	Description
tsiRdata	Interpolates (weekly) cases and (yearly) births and population vectors to the generation time of the disease
twentymeas	Named list of twenty biweekly (IP = 2) time-series from 1944-1964 England
plotdata	Plots the cases, births, and population time series
runtsir	Reconstructs susceptible dynamics, estimates parameters, and runs the forward simulation
plotres	Plots the fitted regressions, reporting, susceptible reconstruction, estimated parameters, and the fit diagnostics
plotcomp	Plots the data versus the simulations only
estpars	Reconstructs the susceptible dynamics and estimates parameters
maxthreshold	Optimizes the threshold parameter for sparse data using *R*^2^
mcmcestpars	Using MCMC, reconstructs susceptible dynamics and estimates parameters
mcmctsir	Using MCMC, reconstructs susceptible dynamics, estimates parameters, and runs the simulation
simulatetsir	Runs the forward simulation taking in the output from estpars or mcmcestpars
plotregression	Plots the cumulative cases and cumulative births regression
plotrho	Plots the inferred reporting rate *ρ*
plotsbar	Plots the inferred $\bar{S}$ and the profile log likelihood
plotbeta	Plots the inferred *β*
plotforward	Plots the data against the mean simulation with confidence intervals

## 2 Conclusions, limitations, and future work

The **tsiR** package allows researchers to fit the time-series Susceptible-Infected-Recovered model using a number of different fitting options that are easy to change and compare. Per the model formulation, the frequentist fitting techniques are computationally tractable and, to a first approximation, work very well for childhood diseases in a number of settings.

However, the model does make a number of assumptions that are undesirable for certain data and pathogens. For example, fixing the infectious period may not be realistic for more chronic infections, and ignoring deaths may lead to biased conclusions when examined over a long enough time scale. Additionally, under certain settings and time scales, the assumption that cumulative cases approximates cumulative births may be flawed. Furthermore, the TSIR model only includes the observation process as a single reporting rate and not as a probability distribution, and stochasticity cannot be explicitly estimated. To include these complexities, methods such as Sequential Monte Carlo and Iterated Filtering can be used to perturb parameters in order to maximize the likelihood [10, 25]. These algorithms are included in the **R** package **pomp** [14]. Such flexibility does comes at a cost, however, as Maximum Likelihood methods can be computationally expensive and optimization algorithms are often complex [11, 14, 26].

Regardless, for fully-immunizing childhood infections such as measles, the TSIR model is able to accurately capture the parameters of interest across a range of different scenarios and remains the most tractable approach (in particular for large numbers of time-series). Improvements and areas of future work to the **tsiR** package include incorporating spatial disease spread (e.g., the gravity model per [18, 27]), the addition of an immigration parameter to the force of infection, a more statistically robust method to estimate initial conditions, and a more streamlined Bayesian approach to the TSIR model. In the spirit of open science, other researchers are welcome to send suggestions, bug reports, as well as contributions to the software.

## Supporting information

S1 File R script. Annotated code to run the analysis in this paper (with additional plots).
(R) Click here for additional data file.
